# Alterations in the gut microbiota and its metabolites contribute to metabolic maladaptation in dairy cows during the development of hyperketonemia

**DOI:** 10.1128/msystems.00023-24

**Published:** 2024-03-19

**Authors:** Zhengzhong Luo, Zhenlong Du, Yixin Huang, Tao Zhou, Dan Wu, Xueping Yao, Liuhong Shen, Shumin Yu, Kang Yong, Baoning Wang, Suizhong Cao

**Affiliations:** 1 College of Veterinary Medicine, Sichuan Agricultural University, Chengdu, China; 2 West China School of Basic Medical Sciences and Forensic Medicine, Sichuan University, Chengdu, China; 3 College of Animal Science and Technology, Chongqing Three Gorges Vocational College, Chongqing, China; California State University, Stanislaus, Turlock, California, USA

**Keywords:** dairy cows, hyperketonemia, multi-omics, gut microbiota, microbial metabolites, metabolic maladaptation

## Abstract

Metabolic maladaptation in dairy cows after calving can lead to long-term elevation of ketones, such as β-hydroxybutyrate (BHB), representing the condition known as hyperketonemia, which greatly influences the health and production performance of cows during the lactation period. Although the gut microbiota is known to alter in dairy cows with hyperketonemia, the association of microbial metabolites with development of hyperketonemia remains unknown. In this study, we performed a multi-omics analysis to investigate the associations between fecal microbial community, fecal/plasma metabolites, and serum markers in hyperketonemic dairy cows during the transition period. Dynamic changes in the abundance of the phyla Verrucomicrobiota and Proteobacteria were detected in the gut microbiota of dairy cows, representing an adaptation to enhanced lipolysis and abnormal glucose metabolism after calving. Random forest and univariate analyses indicated that *Frisingicoccus* is a key bacterial genus in the gut of cows during the development of hyperketonemia, and its abundance was positively correlated with circulating branched-chain amino acid levels and the ketogenesis pathway. Taurodeoxycholic acid, belonging to the microbial metabolite, was strongly correlated with an increase in blood BHB level, and the levels of other secondary bile acid in the feces and plasma were altered in dairy cows prior to the diagnosis of hyperketonemia, which link the gut microbiota and hyperketonemia. Our results suggest that alterations in the gut microbiota and its metabolites contribute to excessive lipolysis and insulin insensitivity during the development of hyperketonemia, providing fundamental knowledge about manipulation of gut microbiome to improve metabolic adaptability in transition dairy cows.

**IMPORTANCE:**

Accumulating evidence is pointing to an important association between gut microbiota-derived metabolites and metabolic disorders in humans and animals; however, this association in dairy cows from late gestation to early lactation is poorly understood. To address this gap, we integrated longitudinal gut microbial (feces) and metabolic (feces and plasma) profiles to characterize the phenotypic differences between healthy and hyperketonemic dairy cows from late gestation to early lactation. Our results demonstrate that cows underwent excessive lipid mobilization and insulin insensitivity before hyperketonemia was evident. The bile acids are functional readouts that link gut microbiota and host phenotypes in the development of hyperketonemia. Thus, this work provides new insight into the mechanisms involved in metabolic adaptation during the transition period to adjust to the high energy and metabolic demands after calving and during lactation, which can offer new strategies for livestock management involving intervention of the gut microbiome to facilitate metabolic adaptation.

## INTRODUCTION

Over production and long-term overwork are important factors contributing to metabolic disorders in livestock. The rising prevalence of metabolic diseases, such as ketosis, milk fever, and fatty liver, seriously limits the production and shortens the lifespan of livestock (1, 2). In particular, recent studies report a high prevalence and incidence of metabolic diseases in dairy cows, and approximately one-third to one-half of early lactation dairy cows are diagnosed with ketosis (1, 3, 4). Hyperketonemia or ketosis is a physiology condition characterized by the elevation of circulating ketones, including acetoacetate, β-hydroxybutyrate (BHB), and acetone (5), which is diagnosed in dairy cows according to a blood concentration of BHB ≥1.2 mmol/L (6, 7). Hyperketonemia is an important contributor to culling in early lactating dairy cows, and can also increase the risk of other diseases such as displaced abomasum, but does not affect milk yield (8–10). Regarding the operational efficiency of farms, the cost of losses from hyperketonemia is higher for primiparous dairy cows than for multiparous cows (11, 12). The pathological mechanism has traditionally been considered to involve the mobilization of body fat in transition dairy cows, which is required to adapt to a high glucose demand after calving, but simultaneously enhances lipolysis and ketogenesis. Thus, failure of appropriate metabolic adaptation during the transition period can lead to hyperketonemia (13, 14). In clinical practice, dairy cows with ketosis typically maintain a high BHB level for a long time without intervention, which elevates the risks for developing other diseases, such as mastitis and displaced abomasum (15, 16). Hyperketonemia in dairy cows occurs more frequently in the first week after calving (17), whereas hyperketonemia diagnosed during the first week of lactation has a negative effect on herd removal and milk yield in dairy cows (18). Ha et al. (19) report and our previous study (20) found that dairy cows experience significant metabolic challenges before hyperketonemia is diagnosed. However, little is known about the dynamic changes in ketogenesis occurring at the different stages of hyperketonemic development.

Accumulating evidence demonstrates that the gut microbiota participate in the development of metabolic disorders (21). However, the majority of research in the field with respect to dairy cows has focused on the microbiota of the rumen, with the role of the gut microbiota largely ignored to date (22). With the extensive use of rumen-protected nutrients, gut microbiota and its functions in dairy cows have attracted increasing attention (23, 24) and several studies have confirmed that gut dysbiosis contributes to the development of diseases in dairy cows (25, 26). Remarkably, the metabolic changes occurring in dairy cows with hyperketonemia were found to impair the growth and gut microbiome development in offspring (27). However, knowledge of the links between the gut microbial community and hyperketonemia in dairy cows remains limited.

The fecal metabolome is regarded as a functional readout of microbial activity, which can be used to explore the relationship between the gut microbiome and host phenotypes (28). Based on analysis of the fecal metabolome, gut microbial metabolites were found to act as metabolic messengers for the regulation of glucose and lipid metabolism (29). For example, lithocholic acid, a bile acid derived from gut microbiota, can stimulate glucagon-like peptide-1 secretion to regulate the glucose metabolism homeostasis by activating aka Takeda G protein-coupled receptor-5 (TGR5) (30, 31). Recent studies demonstrated that the secondary bile acids (SBAs) glycollithocholic acid and taurolithocholic acid are reported to be associated with excessive lipolysis in transition dairy cows (32). Although the composition of bile acids in the different intestinal segments of dairy cows has been reported (33), the association between gut microbial metabolites and metabolic disorders in dairy cows is poorly understood.

Based on this background, we hypothesized that the dynamic changes in gut microbial community are associated with host phenotypes in dairy cows during the transition period and alterations in gut microbial function contributes to the development of hyperketonemia. To investigate the links between the gut microbiota and hyperketonemia, we integrated the longitudinal gut microbial (feces) and metabolic (feces and plasma) profiles to characterize the phenotypic differences between healthy and hyperketonemic dairy cows from late gestation to early lactation. These results are expected to supplement the current research gap on the role of gut microbial function in metabolic adaptation of dairy cows, promote the understanding of the pathogenesis of hyperketonemia, and provide a basis for strategy of health management strategies in dairy herds.

## MATERIALS AND METHODS

### Animals and samples

The experiments were performed at a commercial dairy farm in Sichuan Province, China, from January to April 2022. We selected 20 dairy cows with similar body condition scores, actual days of pregnancy, calf weight, and calving ease scores, including 10 in the healthy (HE) group and 10 in the hyperketonemic (HYK) group, among 90 primiparous cows based on blood BHB concentrations above 1.2 mmol/L at d +7. Details about the cohort selection and evaluation criteria for transitional diseases can be found in our previous studies (20, 34). Fecal and blood samples were collected 1 week before the due date (d –7), within 6 h of calving (d 0), 1 week after calving (d +7), and 2 weeks after calving (d +14) at 0700 before the morning feeding. Blood BHB concentrations of cows were detected at d –7, d 0, d +7, and d +14 using a NovaVat Blood Ketone Meter (WD1621, Nova Bio Vet, Waltham, MA, USA). Serum and plasma samples were collected (using heparin sodium as an anticoagulant), centrifuged at 1,500 × *g* for 10 min at 25°C, and stored at −80°C.

### Serum marker analyses

Serum levels of glucose, insulin, non-esterified fatty acids (NEFA), triglyceride (TG), and total cholesterol were determined using commercially available test kits (Nanjing Jiancheng Bioengineering Institute, China). The revised quantitative insulin sensitivity check index including BHB (RQUICKI_BHB_) was also calculated based on serum levels of NEFA, insulin, glucose, and BHB (35).

### 16S rRNA gene amplicon sequencing of feces and data processing

Total DNA from the stool samples of dairy cows was extracted using the cetyltrimethylammonium bromide method. Amplification of the 16S rRNA gene was performed using primers 341F (5′-CCTACGGGNGGCWGCAG-3′) and 805R (5′-GACTACHVGGGTATCTAATCC-3′) (36). Amplicon pools were assessed and sequenced using a NovaSeq 6000 platform (Illumina, Inc., San Diego, CA, USA). The primer sequences of the raw data were removed using Cutadapt software (v.4.4, https://cutadapt.readthedocs.io/en/stable). Paired-end reads were merged using the FLASH package (v.1.2.11) in R software. The low-quality 3′ regions in reads were trimmed by the fqtrim package (v.0.9.7, http://ccb.jhu.edu/software/fqtrim), and the trimming of chimera was performed using the VSEARCH package (v.2.23). After length filtering and denoising, amplicon sequence variants (ASV)/features were obtained using DADA2 in the QIIME2 (https://qiime2.org) pipeline. The annotations of species in amplicon sequence variants were analyzed on the SILVA database (https://www.arb−silva.de) with the annotated threshold set to 0.7. The microbiome function was predicted using PICRUSt2 (37).

### Fecal metabolome analysis and data processing

After sample pre-treatment, fecal metabolome profiling was performed using ultra-high-performance liquid chromatography (UHPLC; Agilent 1290 Infinity II, Santa Clara, CA, USA) with tandem mass spectrometer (MS/MS; Triple Time-of-Flight 6600, AB SCIEX, Framingham, MA, USA). The pre-treatment conditions and equipment parameters are described in our previous report (38). The mass/charge ratio, retention time, and peak area of the metabolomic features were extracted using the XCMS (https://xcmsonline.scripps.edu) platform. Metabolic features identification was performed using MetabolitePilot software (v.2.0, AB SCIEX, Framingham, MA, USA). The MS/MS spectra were matched with an in-house database (Shanghai Applied Protein Technology) based on retention time, accurate mass (<10 ppm), and spectral patterns. Metabolites in the feces were confirmed based on a Metabolomics Standards Initiative (MSI) level 1 or 2 (39).

After the metabolomic data were processed using log_10_ transformation and Pareto scaling, principal component analysis and orthogonal partial least-squares discriminant analysis (OPLS-DA) were performed using the Ropls package in R. A variable importance in projection >1 from the OPLS-DA model and *P* < 0.05 were set as thresholds for screening differential metabolites between groups. The origin of the metabolites, including hosts and microbes, was analyzed using MetOrigin (40).

### Targeted analysis of plasma metabolites

A 200 µL plasma sample was mixed with 800 µL acetonitrile:methanol (1:1, vol/vol) containing deuterated internal standards to compensate for ionization losses. All mixtures were sonicated in an ice-water bath for 30 min, and incubated at −20°C for 60 min, and then centrifuged at 14,000 × *g* for 20 min at 4°C. The supernatant was collected and dried under a vacuum. A total of 100 µL acetonitrile:water (1:1, vol/vol) was added, mixed, and centrifuged at 14,000 × *g* for 15 min at 4°C. The supernatant was separated by UHPLC (1290 Infinity II, Agilent Technologies) incorporating a BEH C18 column (1.7 µm, 2.1 mm × 100 mm; Waters, Milford, MA, USA) or Amide column (3.5 µm, 4.6 mm× 150 mm; Waters) with an injection volume of 2 µL, flow rate of 0.4 mL/min, and the column temperature maintained at 40°C. The mobile phase and gradient elution procedure for UHPLC are described in our previous report (20). Mass spectrometry analysis was performed using a QTRAP 6500 mass spectrometer (AB SCIEX, Macclesfield, UK). MS/MS data were collected using a multiple reaction monitor and the peak area was acquired using the MultiQuant software (v.3.0.3; AB SCIEX).

The structures of amino acids (AAs) and bile acids were identified by comparison with standards (Additional File 1; Tables S1 and S2). The levels of the targeted metabolites were calculated based on the calibration curves and corresponding regression coefficients. Based on their metabolic pathways, AAs were divided into glucogenic, ketogenic, and glucogenic and ketogenic AAs. Bile acids were defined as 12-OH and non-12-OH bile acids based on presence of the 12-carbon group (Additional File 2; Fig. S1).

### Statistical analysis

Based on the changing characteristic of BHB levels in HYK dairy cows, the time points were categorized into two periods: the non-hyperketonemia period (from d −7 to d 0) and the hyperketonemia period (from d +7 to d +14). Time-series analysis of the serum variables and gut microbiota in dairy cows from late gestation to early lactation was performed using the SantaR package in R. Differences between the HE and HYK groups at a given time were analyzed using the Wilcoxon rank-sum test or Student’s *t*-test as appropriate. Fecal microbiota diversity, including Chao1 and Shannon indices, was analyzed using the vegan package in R. To evaluate the difference in heterogeneous community structure between the HE and HYK groups, principal coordinate analysis was performed based on Bray-Curtis dissimilarity using the vegan package, followed by adonis analysis with 999 permutations. The enterotypes of bacterial patterns at different time points were analyzed using the ade4 and cluster packages based on between-class analysis. Co-occurrence among the genera was evaluated using the MicrobiomeAnalyst platform (https://www.microbiomeanalyst.ca) (41) based on the SparCC algorithm. To screen for important microbial taxa during the transition period, random forest analysis and receiver operating characteristic curve analyses were performed using the randomForest and multipleROC packages, respectively. Spearman’s correlation coefficient was computed to analyze the relationship between the metabolites and microbes using the stats package. Analyses of the matrix between microbiota and blood variables were performed using the Mantel test in the vegan package. Graphics were generated using R tools and GraphPad Prism software (version 10.0).

## RESULTS

### Dynamic changes of serum variables in dairy cows with hyperketonemia from late gestation to early lactation

**Fig 1:**
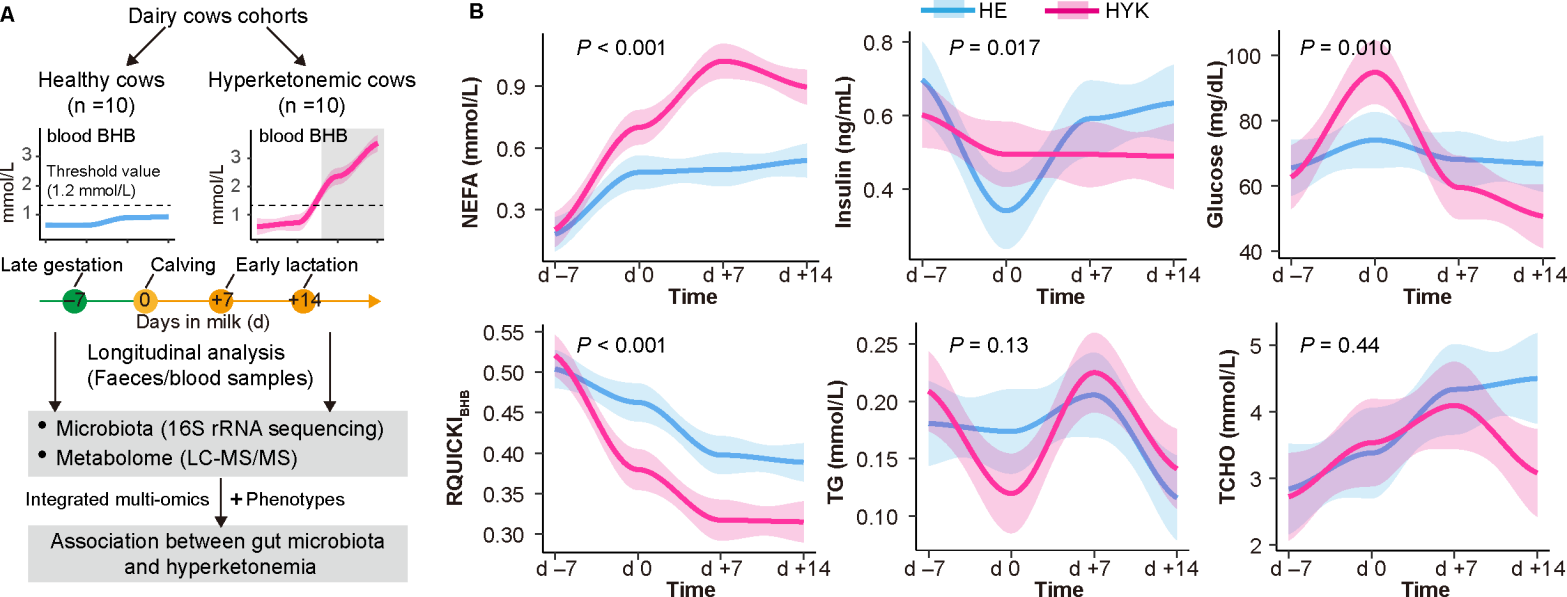
Establishment of two cohorts of dairy cows. (**A**) Overview of the study design. (**B**) Dynamic changes of serum variables in HE and HYK dairy cows from late gestation (d −7) to early lactation (d +14). TCHO, total cholesterol; LC-MS/MS, liquid chromatography-tandem mass spectrometry.

The overall study design is schematically presented in Fig. 1A. Cows with a blood BHB concentration maintained under 1.2 mmol/L were assigned to the HE group, whereas cows showing an increase in the BHB concentration passing above the threshold of 1.2 mmol/L during lactation were assigned to the HYK group.

Compared to that of the HE group, the NEFA level in the HYK group significantly increased after calving (Fig. 1B). Before the hyperketonemia period, at d 0, the levels of insulin, glucose, and TG were significantly different between the HE and HYK groups. Of note, RQUICKI_BHB_ decreased continuously in the HYK group from d 0 to d +14 compared to that in the HE cows. During the hyperketonemia period, glucose and cholesterol levels in the HYK group decreased significantly at d +14 compared to those in the HE group. However, the levels of glucose, insulin, TG, and total cholesterol were not significantly different between the HE and HYK groups on d +7.

### Longitudinal changes in the gut microbial community are related to the metabolic adaptation of transition dairy cows

**Fig 2:**
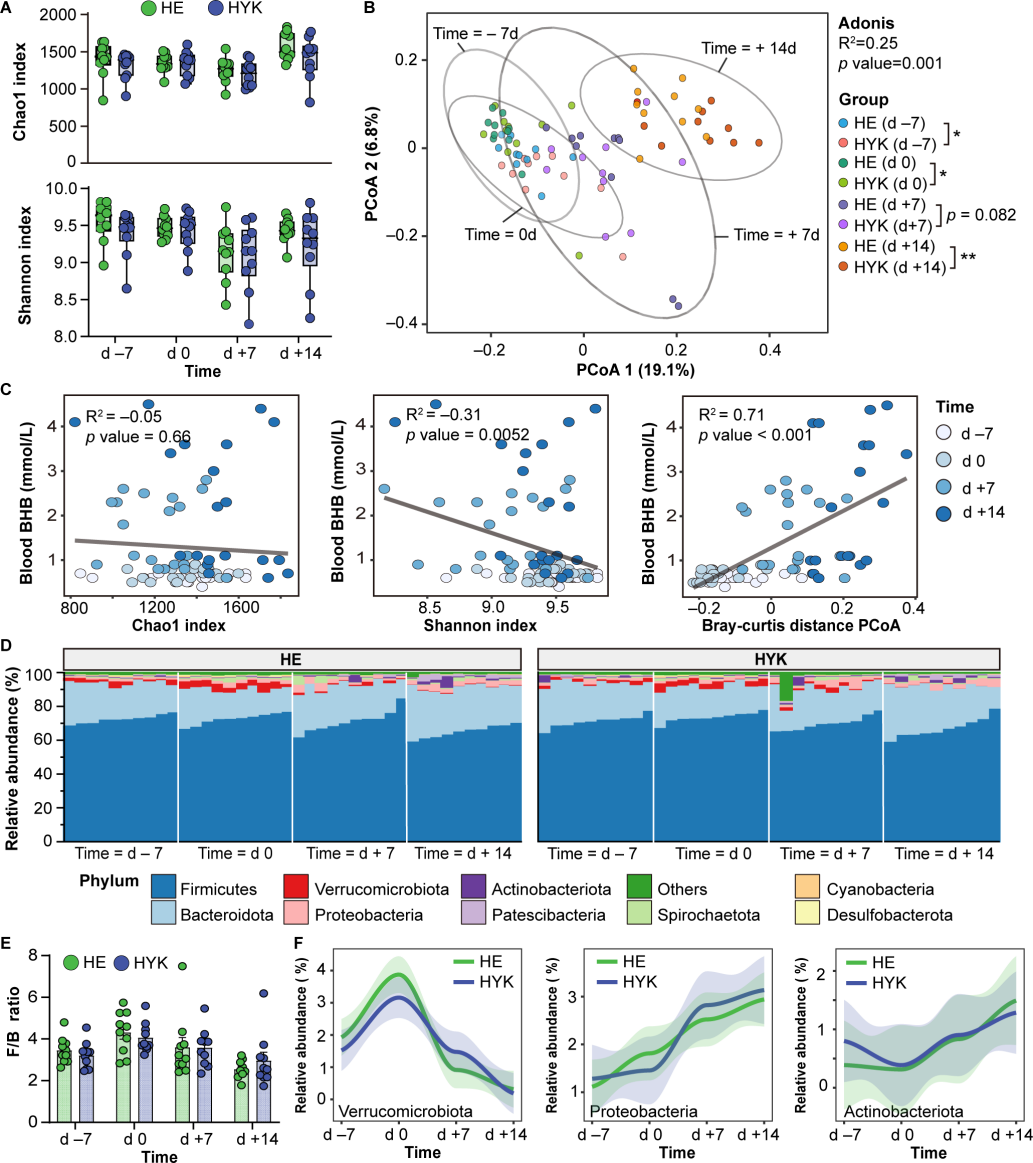
Longitudinal changes of the fecal microbial community in HE and HYK dairy cows. (**A**) Alpha-diversity of the fecal microbiota, including Chao1 and Shannon indices, at different time points. (**B**) Principal coordinates analysis (PCoA) plot of beta-diversity based on the Bray-Curtis distance. (**C**) Associations between blood BHB level and microbial diversity index according to Spearman’s rank correlation coefficient. (**D**) Percentage accumulation chart displaying the relative abundance of microbial taxa at the phylum level. (**E**) Ratio of Firmicutes to Bacteroidota (F/B) within and between the HE and HYK cows at each time point. Data are presented as means ± SEM. (**F**) Dynamic changes of relative abundance of the microbial phyla Verrucomicrobiota, Proteobacteria, and Actinobacteriota. *P < 0.05. **P < 0.01.

The richness and evenness indices of the fecal microbiota, including the Chao1 and Shannon diversity indices, did not differ significantly between the HE and HYK groups at any time point (Fig. 2A). However, the composition of the gut microbial communities in cows were clearly altered (adonis *r* = 0.25, *P* = 0.001) from d −7 to d +14 (Fig. 2B). Moreover, the community structure of the gut microbiota was significantly different between the HE and HYK groups at d −7, d 0, and d +14. However, the microbial communities were not significantly altered in the HYK cows on d +7.

To further investigate the changes in the community structure of gut microbiota, enterotype analysis of dairy cows during the transition period was performed. Based on enterotype-like clusters, we observed that the cows had three enterotypes from late gestation to early lactation, with cows at d −7 and d 0 classified as enterotypes 1 (Additional File 2; Fig. S2). Nevertheless, HE and HYK cows showed similar enterotypes at any given time. To identify correlations between the gut microbiota and hyperketonemia, we performed a correlation analysis for gut microbial diversity and changes in the blood BHB level. The increase in blood BHB level was negatively correlated (*r* =−0.31, *P* = 0.0052) with the Shannon index and was positively correlated (*r* = 0.71, *P* < 0.001) with the composition of the microbial communities (Fig. 2C).

At the phylum level, Firmicutes, Bacteroidetes, Verrucomicrobiota, and Proteobacteria were highly abundant in the feces of dairy cows from late gestation to early lactation and did not differ between the HE and HYK groups at any time point (Fig. 2D; Additional File 1; Table S3). The Firmicutes to Bacteroidota ratio was also not significantly different between the groups (Fig. 2E). Notably, the abundance of Verrucomicrobiota decreased continuously after calving, whereas the abundances of Proteobacteria and Actinobacteriota increased from d 7 to d +14 (Fig. 2F). In addition, the abundance of Verrucomicrobiota was strongly negatively correlated with the level of circulating BHB (*r* = −0.46, *P* < 0.001) and was positively correlated with RQUICKI_BHB_ (*r* = 0.34, *P* < 0.01) and glucose (*r* = 0.33, *P* < 0.01) levels (Additional File 2; Fig. S3). In contrast, the abundance of Proteobacteria was strongly positively correlated with circulating BHB (*r* = 0.57, *P* < 0.001) and NEFA (*r* = 0.44, *P* < 0.001) levels and negatively correlated with RQUICKI_BHB_ (*r* =−0.54, *P* < 0.001) level.

**Fig 3:**
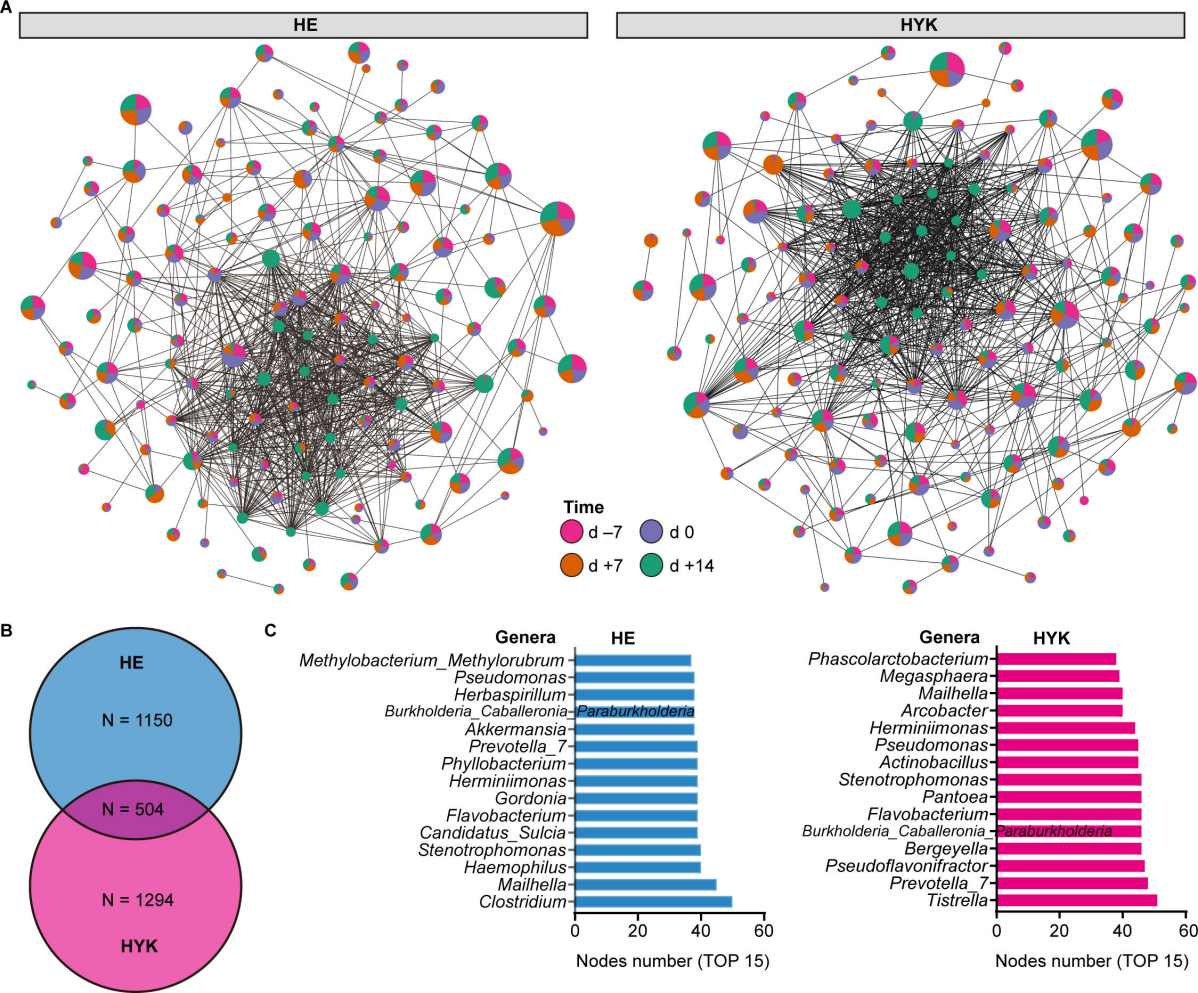
Co-occurrence network analysis of gut microbiota. (**A**) The co-occurrence networks of genera in HE and HYK dairy cows during d −7 to d +14. Only genera with a correlation coefficient >0.5 or <−0.5 and significance of *P* < 0.05 are shown. The node size indicates the proportion of mean abundance in the genus and the node color represents the relative abundance in different phases. (**B**) Venn diagram indicating the edge numbers in each network and their overlap. (**C and D**) Histograms displaying the edge numbers for the top 15 microbial taxa of HE (blue) and HYK (pink) cows, respectively, at the genus level.

To further investigate the dynamic community structure of gut microbiota, we identified 130 genera in the HE and HYK cows that were enriched in the co-occurrence networks (Fig. 3A). However, only 99 shared genera taxa between the HE and HYK groups were observed in the co-occurrence relationships, indicating distinct co-occurrence patterns for the two groups of dairy cows during the transition period. In the gut microbiota of HYK cows, 1,798 connections were found, with most relationships including *Tistrella*, *Prevotella*_7, *Pseudoflavonifractor*, and *Bergeyella* (Fig. 3B and C). In contrast, 1,654 connections were observed in HE cows, with the most common relationships observed among the genera *Clostridium*, *Mailhella*, *Haemophilus*, and *Stenotrophomonas*.

### Altered abundance of gut microbiota is associated with abnormal AA metabolism and enhanced ketogenesis

**Fig 4:**
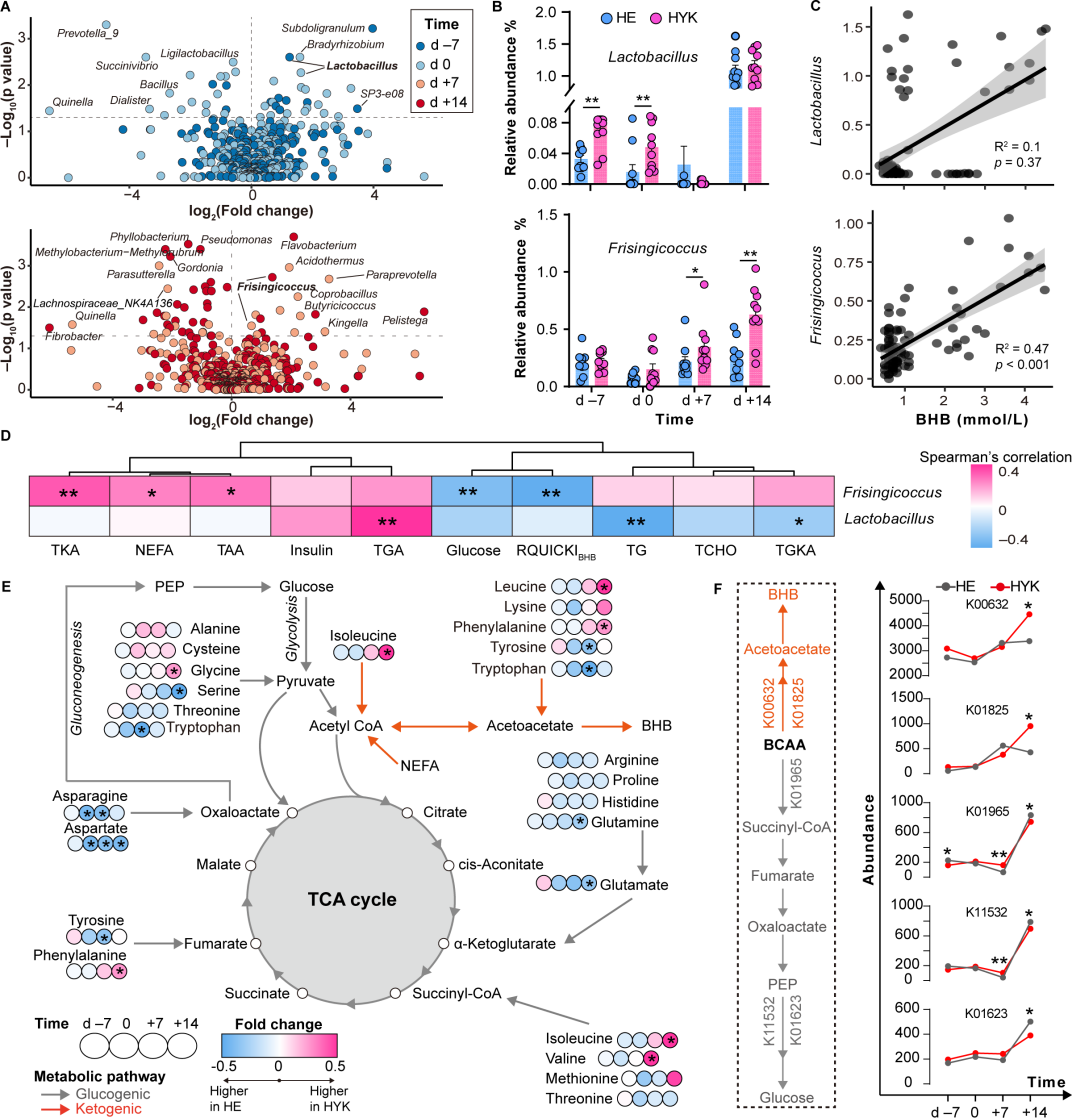
Links between gut microbiota and ketogenesis in dairy cows. (**A**) Volcano plot indicating the differences in the abundances of gut bacteria at the genus level between the HE and HYK cows at each time point. (**B**) Comparisons of *Lactobacillus* and *Frisingicoccus* abundances between the HE and HYK cows over time. Data are presented as means ± SEM. (**C**) Associations between the abundance of *Lactobacillus* or *Frisingicoccus* and circulating BHB levels in dairy cows based on Spearman’s correlation analysis. (**D**) The heatmap displays the associations between serum marker level and the abundance of *Lactobacillus* or *Frisingicoccus*. TKA, total ketogenic amino acids; TGA, total glucogenic amino acids; TGKA, total both glucogenic and ketogenic amino acids; TAA, total amino acids; TCHO, total cholesterol. (**E**) Dynamic change of circulating individual amino acids involved in the glucogenic and ketogenic pathways. The circle color indicates the fold change between HE and HYK groups at each time point. The line color indicates the metabolic pathway of amino acids. PEP, phosphoenolpyruvate; TCA, tricarboxylic acid. (**F**) Metabolism pathway of branched-chain amino acids (BCAAs), involving key Kyoto Encyclopedia of Genes and Genomes orthologs (KO) of microbial taxa related to glucogenesis and ketogenesis. The line chart indicates the dynamic changes of KO abundance between the HE and HYK groups from d −7 to d +14. k00632, fadA/fadI (acetyl-CoA acyltransferase); k01825, fadB (3-hydroxyacyl-CoA dehydrogenase); k01965, pccA (propionyl-CoA carboxylase alpha chain); k11532, glpX-SEBP (fructose-1,6-bisphosphatase II/sedoheptulose-1,7-bisphosphatase); k01623, ALDO (fructose-bisphosphate aldolase, class I). 0.01 < *P < 0.05, **P < 0.01.

In the non-hyperketonemia period, the abundances of *Subdoligranulum* and *Rikenellaceae SP3−e08* were significantly increased in the HYK group at d −7 compared with those of the HE group (Fig. 4A; Additional File 1; Table S4). The abundances of *Prevotella_9*, *Succinivibrio*, *Ligilactobacillus, Bacillus*, *Dialister*, and *Quinella* decreased significantly in the HYK group at d 0. During the hyperketonemia period, the abundances of *Acidothermus*, *Paraprevotella*, *Coprobacillus*, and *Bradyrhizobium* were increased, whereas the abundances of *Lachnospiraceae_NK4A136_group*, *Parasutterella*, and *Quinella* markedly decreased in the HYK group at d +7 compared to those of the HE group. In addition, the abundances of *Flavobacterium*, *Pelistega*, and *Butyricicoccus* in HYK cows on d +14 were higher in the HYK group than in HE cows.

Of note, the abundance of *Lactobacillus* was markedly increased in the HYK group on d −7 and d 0 compared with that in the HE group at the corresponding time points (Fig. 4B). However, blood BHB levels were not significantly correlated with the abundance of *Lactobacillus* (Fig. 4C). Compared with that of HE cows, the abundance of *Frisingicoccus* was increased in the HYK group on d +7 and d +14 and was positively correlated (*r* = 0.47, *P* < 0.001) with blood BHB levels. Random forest analysis indicated that *Frisingicoccus,* belonging to the phylum *Firmicutes*, was an important contributor to the development of hyperketonemia (Additional File 2; Fig. S4).

To investigate the relationship between gut microbes and ketogenesis, we performed an association analysis among key genera, metabolic phenotypes, and serum AAs. The abundance of *Frisingicoccus* was positively correlated with the levels of total ketogenic AAs, NEFA, and total AAs, and was negatively correlated with the levels of glucose and RQUICKI_BHB_ (Fig. 4D). Moreover, the abundance of *Lactobacillus* was positively correlated with total glucogenic AA levels and was negatively correlated with both total glucogenic and ketogenic AA levels. The levels of circulating glucogenic AAs, including arginine, asparagine, glutamic acid, glutamine, and serine, were markedly decreased in HYK cows on d +14 compared to those in HE cows at the same time point (Fig. 4E). However, branched-chain AAs (BCAAs), including leucine, isoleucine, and valine, increased significantly in HYK cows on d +14 and were strongly positively correlated with an increase in the abundance of *Frisingicoccus* (Additional File 2; Fig. S5A). In addition, the levels of BCAAs were strongly correlated with the levels of circulating BHB and glucose (Additional File 2; Fig. S5B).

We further analyzed the changes in the gut microbiota genes encoding enzymes in the BCAA metabolism pathway from late gestation to early lactation (Additional File 1; Table S5). The ketogenic-related genes, including acetyl-CoA acyltransferase (k00632) and 3-hydroxyacyl-CoA dehydrogenase (k01825), were upregulated in the HYK group on d +14 (Fig. 4F), whereas the glucogenesis-related genes, including propionyl-CoA carboxylase alpha chain (k01965), fructose-1,6-bisphosphatase II (k11532), and fructose-bisphosphate aldolase class I (k01623), were downregulated in HYK cows on d +14. Of note, the expression of k01965 and k11532 was significantly upregulated in HYK cows on day +7 compared to that in HE cows.

### Altered fecal metabolites of dairy cows with hyperketonemia

**Fig 5:**
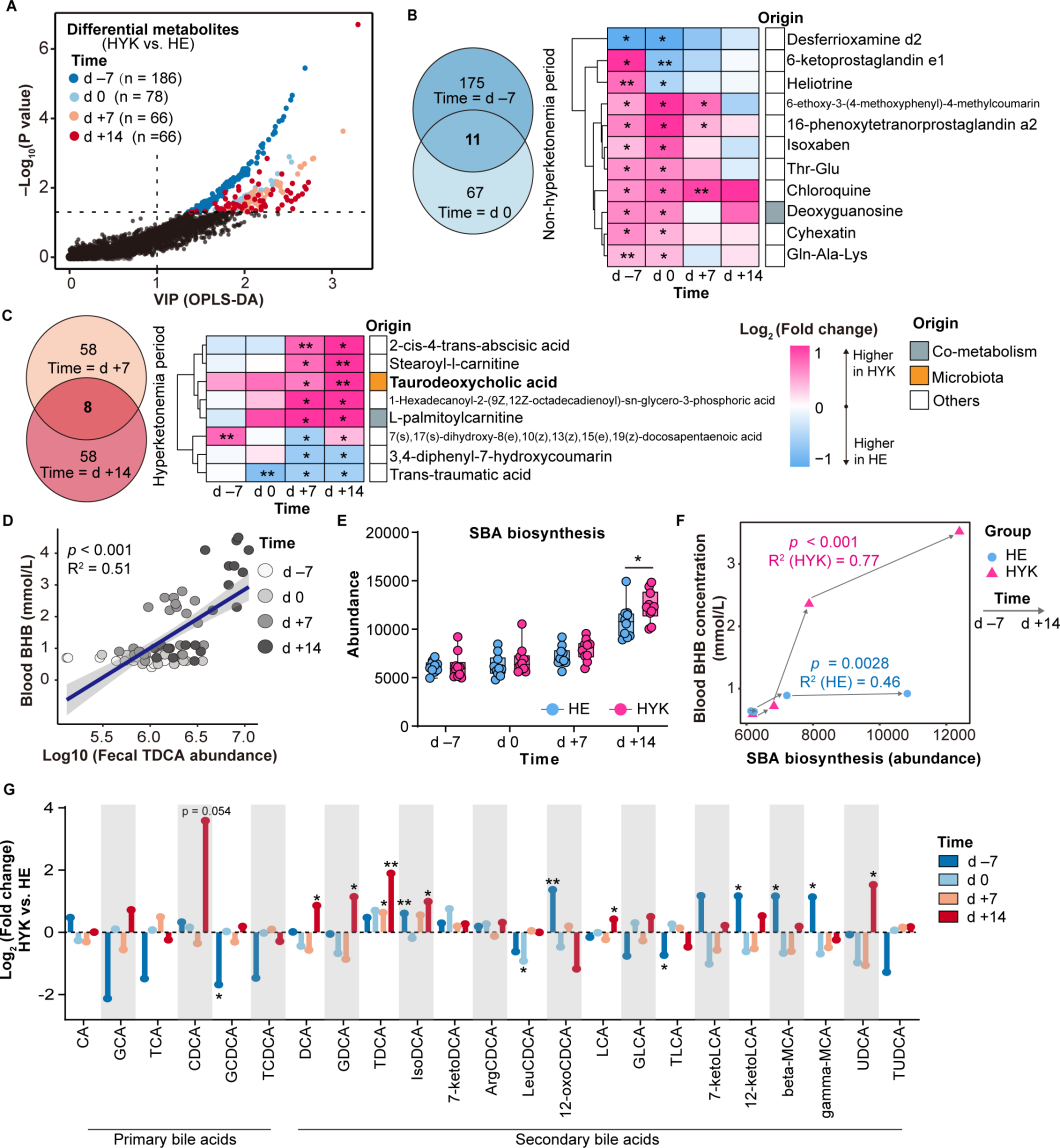
Alterations in fecal metabolites between HE and HYK dairy cows. (**A**) Scatter plot showing differential metabolites between the HYK and HE groups at each time point based on the variance in projection (VIP) value of OPLS-DA. (**B and C**) Change and origin of shared differential metabolites in the non-hyperketonemia and hyperketonemia periods, respectively. (**D**) Associations of blood BHB level with the abundance of fecal TDCA. (**E**) Differential analysis of secondary bile acid (SBA) biosynthesis between HE and HYK cows based on functional prediction of gut microbiota. (**F**) Change of SBA biosynthesis correlated with an increase in the circulating BHB level of dairy cows from late gestation to early lactation. (**G**) Lollipop plot displaying the differences in the levels of individual bile acids in feces of cows in the HE and HYK groups at each time point. 0.01 < *P < 0.05, **P < 0.01.

To determine the role of gut microbial function in the development of hyperketonemia, we performed fecal metabolic profiling. A total of 1091 metabolites were identified at MSI level 1 or 2 (Additional File 2; Fig. S6A; Table S6). A distinct difference in the fecal metabolome profiles was detected between the non-hyperketonemia and hyperketonemia periods (Additional File 2; Fig. S6B). Fold-change analysis showed an increase in the level of fecal BHB over the course of hyperketonemia development in HYK cows (Additional File 2; Fig. S6C). Furthermore, the fecal concentration of BHB was found to positively correlate with the circulating levels of BHB in the blood (*r* = 0.39, *P* < 0.01). Differential analysis of metabolites between the HYK and HE groups at each time point (Fig. 5A) identified 11 and 8 shared metabolites during the non-hyperketonemia and hyperketonemia period, respectively (Fig. 5B and C). In comparison to the HE group, the level of deoxyguanosine significantly increased during the non-hyperketonemia period, while the level of L-palmitoylcarnitine significantly increased during the hyperketonemia period in the hyperketonemic group. Notably, the level of taurodeoxycholic acid (TDCA) derived from the gut microbiota was markedly increased in HYK cows on d +7 and d +14 compared to that in HE cows (Fig. 5C) and the increase in blood BHB level was positively correlated with the increase in fecal TDCA levels (*r* = 0.51, *P* < 0.001; Fig. 5D). Microbial function prediction showed that SBA biosynthesis was markedly upregulated in HYK cows on d +14 (Fig. 5E), which was also strongly and positively correlated with the increase in circulating BHB level (Fig. 5F).

To further investigate the association between bile acids and hyperketonemia, 23 bile acids were identified in the feces of dairy cows (Fig. 5G). Compared with that in the HE cows, the level of the primary bile acid glycol-chenodeoxycholic acid (CDCA) was decreased in the HYK cows on d −7. The levels of some SBAs in the HYK cows, including iso-deoxycholic acid (DCA), 12-oxoCDCA, 12-keto-lithocholic acid (LCA), and beta- and gamma-muricholic acid, were higher than those in the HE cows on d −7. During the hyperketonemia period, SBAs including DCA, glyco-DCA, TDCA, and iso-DCA increased significantly in HYK cows on d +14.

### Abnormal bile acids metabolism is associated with hyperketonemia development

**Fig 6:**
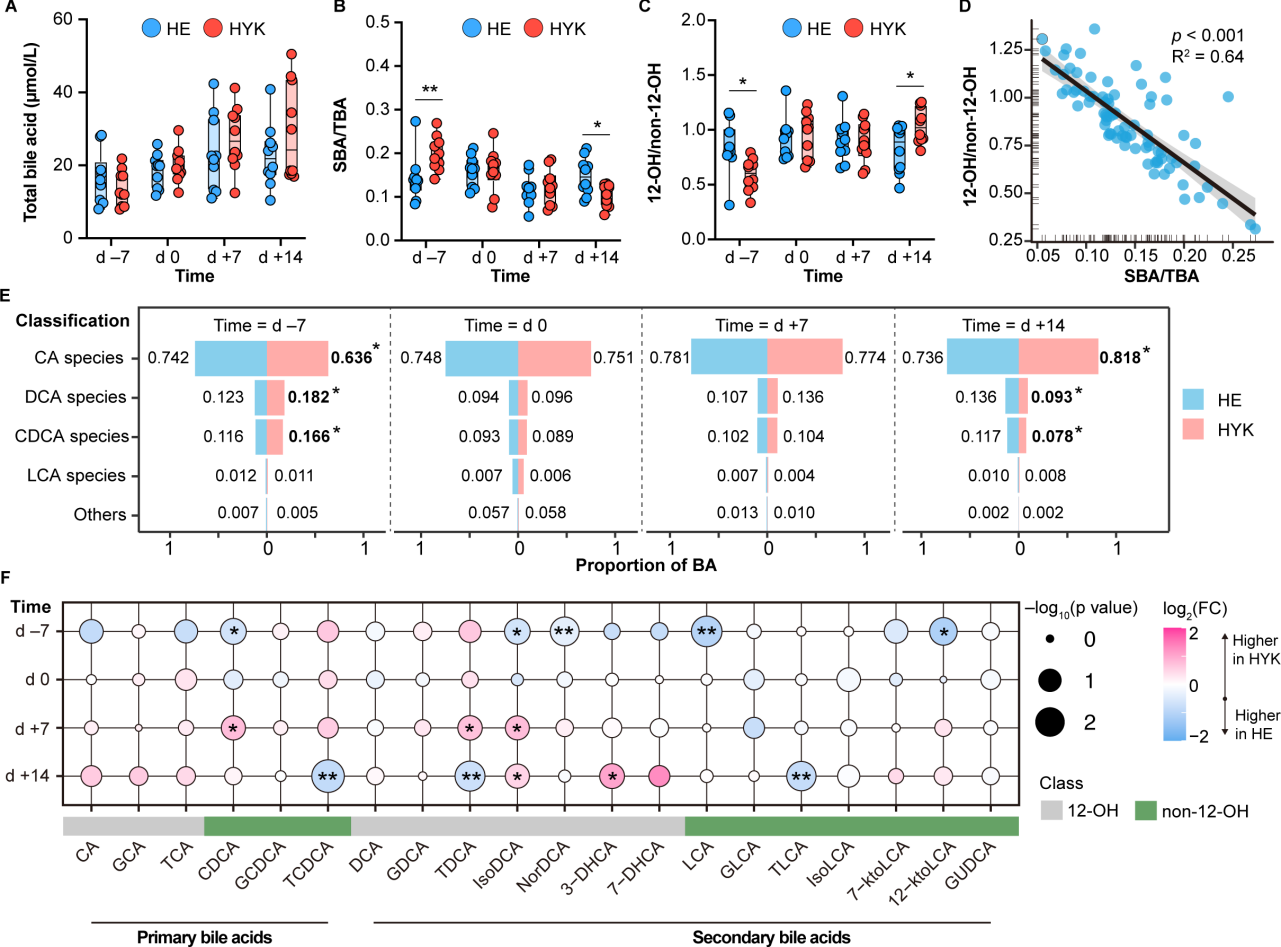
Plasma bile acid (BA) profiles in the HE and HYK dairy cows. (**A**) TBA. (**B**) Ratio of SBA to TBA. (**C**) Ratio of 12-OH to non-12-OH BAs (log_10_-transformed data). (**D**) Associations of 12-OH/non-12-OH BAs with SBA/TBA based on Spearman’s correlation analysis. (E) The classification of BA differences in the HE and HYK groups at different time points. Cholic acid (CA) species comprise CA, glycolic acid (GCA), and taurocholic acid (TCA). DCA species comprise DCA, glycodeoxycholic acid (GDCA), TDCA, isoDCA, and norDCA. Chenodeoxycholic acid (CDCA) species comprise CDCA, glycochenodeoxycholic acid (GCDCA), and taurochenodeoxycholic acid (TCDCA). Lithocholic acid (LCA) species comprise LCA, glycolithocholic acid (GLCA), taurolithocholic acid (TLCA), isoLCA, 7-ketoLCA, and 12-ketoLCA. Other BA species include glycoursodeoxycholic acid (GUDCA), 3-dehydrocholic acid (DHCA), and 7-DHCA. (**F**) Comparison of plasma levels of individual BAs between the HE and HYK groups at each time point. The node size indicates the *P*-value after −log_10_ transformation. The node color indicates the fold change (FC) of individual BA levels between the HE and HYK cows; a log_2_(FC) greater than 0 indicates relatively higher concentration in the HYK cows, whereas a log_2_(FC) less than 0 indicates a lower concentration in HYK cows than in HE cows. 0.01 < *P < 0.05, **P < 0.01.

Twenty types of bile acids were identified in primiparous dairy cows during the transition period, including 10 12-OH and 10 non-12-OH bile acids (Additional File 1; Table S2). The total bile acid (TBA) levels in the plasma from d –7 to d +14 did not differ between the HE and HYK groups (Fig. 6A). However, the ratio of SBA to TBA significantly increased on d −7 and decreased on d +14 in HYK cows compared with that in HE cows (Fig. 6B). By contrast, the ratio of 12-OH to non-12-OH bile acids significantly decreased on d −7 and increased on d +14 in HYK cows (Fig. 6C). Moreover, the SBA-to-TBA ratio was strongly positively correlated (*r* = 0.64, *P* < 0.001) with the 12-OH to non-12-OH bile acid ratio (Fig. 6D).

The main bile acids in primiparous dairy cows belonged to the cholic acid (CA), DCA, CDCA, and LCA species (Fig. 6E). Compared to that in HE cows, the percentage of CA species in HYK cows showed a significant decrease at d –7, whereas the percentages of DCA and CDCA species increased significantly. However, at d +14, the proportion of CA species increased while the proportions of DCA and CDCA species decreased in HYK cows. In addition, the percentages of CA, CDCA, DCA, and LCA species did not differ significantly between HE and HYK cows on d 0 and d +7. In terms of individual bile acids, the levels of isoDCA, norDCA, LCA, and 12-ketoLCA in HYK cows showed a significant decrease on d –7 compared to those in HE cows (Fig. 6F). However, isoDCA levels increased in HYK cows during hyperketonemia. The levels of taurine-conjugated bile acids in the HYK cows on d +14, including taurochenodeoxycholic acid (TCDCA), TLCA, and TDCA, were lower than those in HE cows.

**Fig 7:**
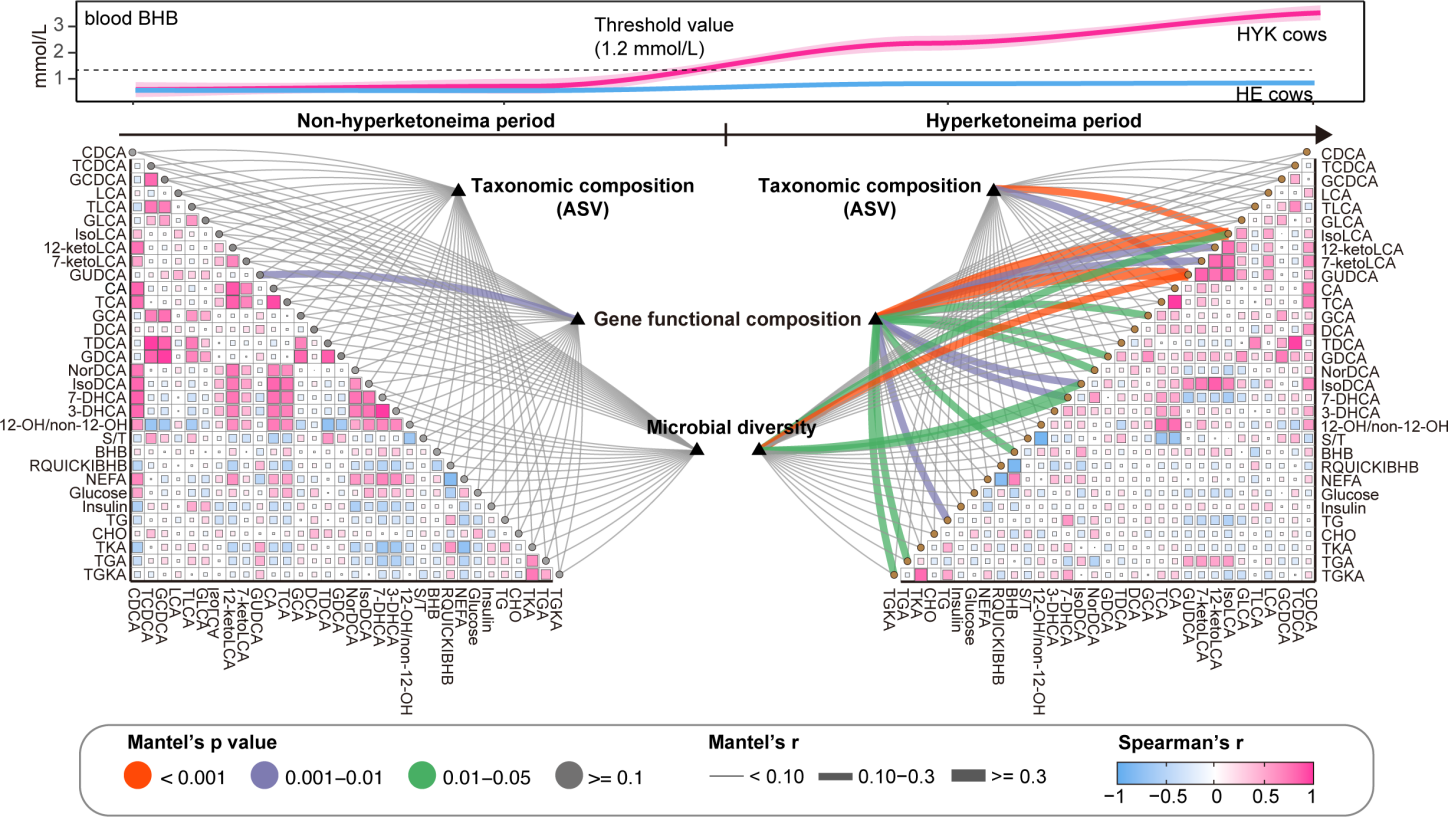
Links between gut microbial community and host phenotypes in non-hyperketonemia and hyperketonemia periods. The heatmap displays the relationships among serum markers, plasma bile acids, and plasma amino acids composition based on Spearman’s correlation analysis. The line indicates the relationship of the gut microbiota matrix with the blood variables matrix based on the Mantel test. The line color indicates the *P*-value in the Mantel test and the thickness of the line indicates the correlation coefficient. GLCA, glycollithocholic acid; TCA, taurocholic acid; GCA, glycolic acid; GDCA, glycodeoxycholic acid; S/T, secondary bile acids/total bile acids ratio; CHO, cholesterol; TKA, total ketogenic amino acids; TGA, total glucogenic amino acids; TGKA, total glucogenic and ketogenic amino acids.

To further investigate the role of the gut microbiota in hyperketonemia, we performed network association analyses among microbial variables, metabolic markers, and bile acids. We observed that the taxonomic composition and microbial diversity in dairy cows were not associated with circulating metabolic markers during the non-hyperketonemia period (Fig. 7). The gene functional composition of the gut microbiota was strongly correlated with glycoursodeoxycholic acid (GUDCA) levels in the plasma during the non-hyperketonemia period. However, the composition and function of the microbiota were strongly correlated with bile acid metabolism and ketogenesis in dairy cows with hyperketonemia. During hyperketonemia, the functional composition of the gut microbiota was associated with the levels of circulating BHB, total glucogenic and ketogenic AAs, total glucogenic AAs, and SBAs. In addition, microbial diversity strongly correlated with the levels of SBAs, including GUDCA, isoLCA, 7-hyodeoxycholic acid, and isoDCA.

## DISCUSSION

Dairy cows routinely suffer from challenge of metabolic disorders during the transition period, with alteration in host phenotypes most often occurring in the early lactation phase (14, 42). However, the endogenous causes of these metabolic disorders remain unknown. The gut microbiota is considered to be a contributing factor in the development of metabolic diseases. In this study, we confirmed that cows underwent the excessive lipid mobilization and insulin insensitivity before hyperketonemia was evident. In HYK cows, we observed changes in the gut microbial communities during the development of hyperketonemia. The diversity of gut microbiota and the biosynthesis of SBA were linked to elevated levels of circulating BHB. The circulating BCAA levels in HYK cows increased during the hyperketonemia period and microbial gene prediction showed that the enrichment of enzymes in the ketogenic pathway was upregulated concurrently in ketogenesis. Therefore, our results suggest that bile acids are functional readouts that link the gut microbiota and host phenotypes in the development of hyperketonemia. These findings provide novel evidence that gut microbes contribute to hyperketonemia during the transition period in dairy cows.

Poor adaptation to the transition period is often considered to cause hyperketonemia, in which dairy cows exhibit excessive lipid mobilization and high circulating BHB level after calving (14, 43). In the present study, we found that the serum levels of NEFA, glucose, and insulin were increased in HYK cows at the calving date. In line with our findings, previous studies also reported that increased NEFA levels in HYK dairy cows were accompanied by an increased BHB concentration (19, 44). In a poor adaptation state, abundant NEFA are released from the adipose tissue, and further metabolized to TG or BHB in the liver (13). However, the accumulation of TG occurs earlier than BHB production, which can explain the increase in the BHB concentration at 1 week after calving (45). Increased circulating NEFA levels were reported to be associated with the reduced insulin sensitivity in dairy cows with hyperketonemia (46). NEFA can activate the nuclear factor kappa B (NF-κB) pathway by stimulating Toll-like receptor-4 to ultimately inhibit the insulin signaling pathway (47). Shen et al. (48) reported that the NF-κB signaling pathway is over-activated in dairy cows with ketosis. A reduction in insulin sensitivity can also cause increased lipid mobilization, leading to severe metabolic disorders in dairy cows (49). The RQUICKI_BHB_ is typically used to evaluate the insulin sensitivity in dairy cows (35). In the present study, we found that the NEFA, glucose, and insulin levels were elevated, whereas the RQUICKI_BHB_ level was reduced in dairy cows prior to the diagnosis of hyperketonemia, indicating that the pathogenesis of hyperketonemia is associated with higher insulin insensitivity after calving. Therefore, high NEFA and BHB levels may contribute to insulin insensitivity in cows with hyperketonemia. However, we did not use random sampling of dairy herds, which limits inferences on whether excessive lipid mobilization and insulin insensitivity induce the development of hyperketonemia. Thus, further research based on a larger cohort is required to confirm these causal associations.

In recent years, the homeostasis of the gut microbiota community has been recognized as playing a key role in the health and metabolic disease of dairy cows (50). The composition and function of the gut microbial community are closely related with the glucose and lipid metabolism homeostasis (21, 22). A previous study demonstrated that the composition of the gut microbial community in dairy cows was altered to adapt to the high glucose demands after calving (51). Firmicutes, Bacteroidetes, Verrucomicrobiota, Proteobacteria, and Actinobacteria were the primary phyla identified in the hindgut of dairy cows during transition period (51), which is consistent with our present findings. Notably, in contrast to Firmicutes and Bacteroidetes, the abundance of Verrucomicrobiota increased from late gestation to calving, and decreased subsequently during early lactation, while the abundance of Proteobacteria increased rapidly after lactation initiation. The dynamic changes in Verrucomicrobiota and Proteobacteria were consistent with the changes in serum glucose and NEFA levels, respectively. Verrucomicrobiota is regarded as a moderator in the regulation of glucose homeostasis, which is in line with the core metabolic pathways from Verrucomicrobiota functional annotation, such as the glycolysis and gluconeogenesis pathways (52, 53). In dairy cows, increases in lipolysis and ketogenesis contribute to metabolic disorders and inflammation from late gestation to early lactation (54). There is evidence that the gut microbiome acts as an indicators of negative energy balance and inflammation in transition dairy cows (51). By combining the dynamic changes in host phenotype and fecal microbiota, we found that the abundance of Proteobacteria was associated with an increase in metabolic disorders in dairy cows after calving. As the core microbial phylum in the gut, Proteobacteria serves a marker of an unstable microbial community that is often over-represented in metabolic disorders and associated with the development of inflammation (55, 56). Although we did not find significant differences in the abundances of Proteobacteria and Verrucomicrobiota between the HE and HYK cows, these phyla may be the designators of metabolic adaptation in dairy cows from late gestation transit to early lactation, which requires further investigation. In addition, we observed that the richness of the gut microbial community strongly correlated with serum BHB level. In line with our findings, Miles et al. (57) also reported that the alpha-diversity of the gut microbial community was negatively correlated with the circulating NEFA and BHB levels. Therefore, dynamic changes in gut microbial community are associated with the excessive lipolysis and abnormal glucose metabolism in the HYK cows during the transition period.

Previous studies indicated that abnormal AA metabolism may contribute to elevated ketogenesis during the development of hyperketonemia (58, 59). In particular, the levels of circulating BCAAs, including isoleucine, leucine, and valine, were higher in cows undergoing hyperketonemia than in HE cows (58, 60), which is consistent with our current findings. Accumulating evidence suggests that the BCAAs, as gut microbiota-derived metabolites, can induce the insulin resistance by activating mammalian target of rapamycin complex 1 (29, 61). We previously reported that the levels of isoleucine and leucine were markedly increased in dairy cows with left displaced abomasum events and were strongly correlated with the abundances of *Akkermansia* and *Oscillospira* in the feces (38). However, unlike leucine, an increase in isoleucine and valine levels adversely affects metabolic health by disturbing glucose homeostasis (62). In this study, we observed that the metabolic capacity of BCAAs in the ketogenic pathway to bind BHB was elevated in the HYK cows, which was attributed to the associated alterations in the gut microbiota, whereas conversion of isoleucine to glucose in the glucogenic pathway reduced. A previous study has been confirmed that high circulating level of BHB promoted insulin resistance by inducing hepatic endoplasmic reticulum stress in dairy cows (63). In addition, we found that an increase in the abundance of *Frisingicoccus* was strongly correlated with the levels of circulating BCAA and BHB. A nutritional study showed that the abundance of *Frisingicoccus* increased after dietary valine treatment, and long-term high level of valine altered the composition of short-chain fatty acid in the gut (64). *Frisingicoccus,* which belongs to *Lachnospiraceae*, is a butyrate-producing genus associated with the metabolic health (65, 66). In addition, we observed that the butyrate-producing bacterium *Butyricicoccus* was increased in the feces of dairy cows during hyperketonemia period. Although butyrate plays a beneficial role in epithelial cell homeostasis (67), it is metabolized to BHB, and a high dietary level of butyrate is considered a contributing factor to ketosis in dairy cows. Based on our findings, *Frisingicoccus* is a key genus in dairy cows during the development of hyperketonemia; however, whether *Frisingicoccus* participate in the ketogenesis by regulating the metabolism of BCAAs and butyrate needs to be further confirmed. Both BCAAs and butyrate are derived from ruminal microbial catabolism in AA and short-chain fatty acid metabolism (68). Therefore, BCAA is not regarded as an indicator of gut microbial function in dairy cows with hyperketonemia.

To further explain the function of the gut microbial community, we found that bile acids derived from gut microbes altered in the development of hyperketonemia. Unlike other microbial metabolites, SBAs are only metabolized by intestinal microbiota of cows, which act as signaling molecules that participate in the regulation of glycolipid and energy metabolism (69). Primary bile acids are transformed into SBAs via deconjugation, dehydroxylation, oxidation, or epimerization by gut microbiota, including *Bacteroides*, *Bifidobacterium*, *Lactobacillus*, *Ruminococcus*, and *Clostridium* (70). In the present study, we found that the levels of circulating SBAs, including TDCA and TLCA, decreased in cows with hyperketonemia, and this reduction lasted for approximately 1 week. In enteroendocrine cells, LCA and DCA promote glucagon-like peptide-1 secretion by activating TGR5, further increasing insulin sensitivity (71). Furthermore, the levels of circulating LCA and 12-ketoLCA decreased in dairy cows prior to the hyperketonemia event, indicating that the LCA–TGR5 pathway may participate in the regulation of glycolipid metabolism and insulin sensitivity in dairy cows during the transition period, although this hypothesis requires further study.

Based on the association analysis between gut microbial function and bile acids metabolism, we found that GUDCA was an important functional readout involved in lipid mobilization by the gut microbiota in transition dairy cows. A recent study suggested that ursodeoxycholic acid can weaken NEFA-induced insulin resistance via impeding inositol, which is dependent on protein-1α and the protein kinase RNA-like endoplasmic reticulum kinase signaling pathway (72). In addition, we found that the 12-OH:non-12-OH bile acid ratio decreased in HYK cows at the time of diagnosis and then increased during the development of hyperketonemia, lasting for 1 week, and the 12-OH:non-12-OH bile acid ratio was closely correlated with the SBA:TBA ratio. The 12-OH:non-12-OH bile acid ratio has been reported to correlate with metabolic disease and insulin resistance (73). The 12-OH bile acid is mainly derived from the classic bile acid synthetic pathway, in which cholesterol is metabolized into 7α-hydroxycholesterol by the first and limiting enzyme CYP7A1 and is then converted to CA via sterol 12α-hydroxylase (CYP8B1) and CYP27A1 (74). Previous studies have suggested that the adaptation of transition dairy cows to metabolic alterations and the higher energy demands results in enhanced CYP7A1 expression in the liver after calving (75). In addition to the classic bile acid synthetic pathway, there is an alternative pathway in which cholesterol is hydroxylated by the initial enzyme CYP27A1 and is then further metabolized into CDCA by CYP7B1 (74). We found that the level of CDCA, as a non-12-OH primary bile acid, decreased in HYK cows before calving, whereas the level of TCDCA decreased during the hyperketonemia period. In addition, Shahzad et al. (76) reported that the level of glycochenodeoxycholate in liver tissue was altered in cows prior to the diagnosis of hyperketonemia. CDCA is a Farnesoid X receptor (FXR) agonist that modulates lipid and glucose metabolism by activating FXR to induce the production of fibroblast growth factor 21 (77). Hepatic fibroblast growth factor 21 plays an important role in the regulation of metabolic adaptation in dairy cows during the early lactation period (78). Although prepartum exercise has recently been shown to alleviate metabolic stress in dairy cows by elevating the levels of conjugated CDCA during the prepartum period (79), the causality between alterations in bile acid synthesis and hyperketonemia remains unclear and requires further investigation. In particular, we suggest focusing on key regulatory genes in the bile acid synthetic pathway, such as *CYP8B1* and *CYP7B1*, to further elucidate the roles of the classic and alternate synthetic pathways in the development of hyperketonemia.

Overall, our findings indicate that bile acids play a key role in the functional readout linking the gut microbiota and hyperketonemia in dairy cows during the transition period. One limitation of the present study is that it does not confirm the regulatory impact of bile acids on the metabolic adaptation of dairy cows or examine related mechanisms. Future studies should consider dietary supplementation with bile acids to explore whether the rectification of bile acid homeostasis abnormalities can reduce the incidence of hyperketonemia or ketosis in dairy cows during the transition period. It is also suggested to validate the key bacterial species identified in this study using metagenomics and further discern the causality between gut microbes-bile acid axis and metabolic maladaptation in dairy cows.

### Conclusions

Our study identified that dynamic changes in gut microbial taxonomic taxa were correlated with metabolic adaptations in transition dairy cows, including excessive lipid mobilization and insulin insensitivity, whereas abnormal composition of gut microbiota community contributed to the development of hyperketonemia. Furthermore, changes in gut microbial metabolites, such as TDCA, were detected in dairy cows during the hyperketonemic phase, thus establishing a link between the gut microbiota and hyperketonemia. The present study may provide a recommendation for nutritional strategies by manipulating gut microbial homeostasis to facilitate the cow’s successful transition to lactating phase, which helps alleviate the metabolic disorders in periparturient period.

## Data Availability

The 16S rRNA sequences of cattle fecal samples has been deposited into the NCBI Sequence Read Archive (SRA) under the accession numbers PRJNA1032648, PRJNA1032696 and PRJNA1033314.

## SUPPLEMENTAL MATERIAL

Supplemental figures msystems.00023-24-s0001.pdf Figures S1 to S6.

Supplemental tables msystems.00023-24-s0002.xlsx Tables S1 to S6.

ASM does not own the copyrights to Supplemental Material that may be linked to, or accessed through, an article. The authors have granted ASM a non-exclusive, world-wide license to publish the Supplemental Material files. Please contact the corresponding author directly for reuse.
