# Supplementary material for: Alterations in the gut microbiota and its metabolites contribute to metabolic maladaptation in dairy cows during the development of hyperketonemia
Source: mSystems. 2024 Mar 19;9(4):e00023-24. doi: 10.1128/msystems.00023-24 (PMC11019918; doi:10.1128/msystems.00023-24)
Supplement: Supplemental figures — Figures S1 to S6. [file msystems.00023-24-s0001.pdf]

Supplementary Materials

Alterations in the gut microbiota and its metabolites contribute to metabolic maladaptation in dairy cows during the development of hyperketonemia

Zhengzhong Luo,[1,2†] Zhenlong Du,[1†] Yixin Huang,[1†] Tao Zhou, [1] Dan Wu,[1] Xueping Yao,[1] Liuhong Shen,[1] Shumin Yu,[1] Kang Yong [3], Baoning Wang[2*] and Suizhong Cao[1*]

[1]College of Veterinary Medicine, Sichuan Agricultural University, Chengdu 611130, China

[2]West China School of Basic Medical Sciences and Forensic Medicine, Sichuan University, Chengdu 610041, China

[3]College of Animal Science and Technology, Chongqing Three Gorges Vocational College, Chongqing 404100, China

[†]Zhengzhong Luo, Zhenlong Du and Yixin Huang contributed equally to this work.

*Address correspondence to Baoning Wang, wangbn@scu.edu.cn and Suizhong Cao, suizhongcao@sicau.edu

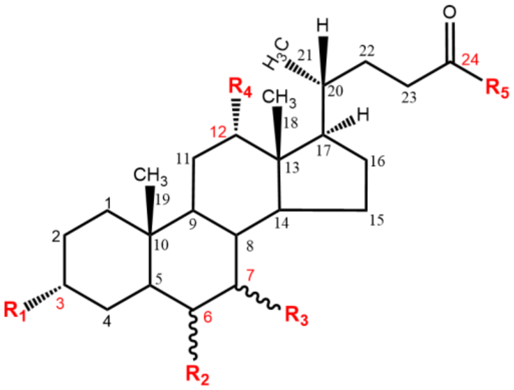

Figure S1. Illustration of chemical structure in bile acid.

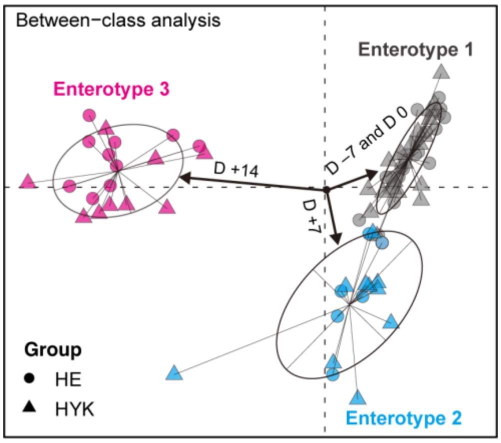

Figure S2. Enterotype analysis between the healthy (HE) and hyperketonemic (HYK) dairy cows from late gestation to early lactation.

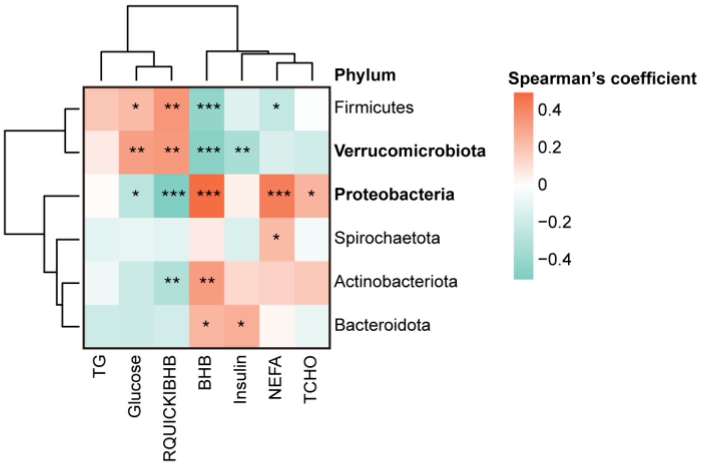

Figure S3. The associations between serum markers and taxonomic composition in phylum level based on Spearman's correlation analysis. ***P < 0.001, 0.001 < **P < 0.01, 0.01 < *P <0.05.

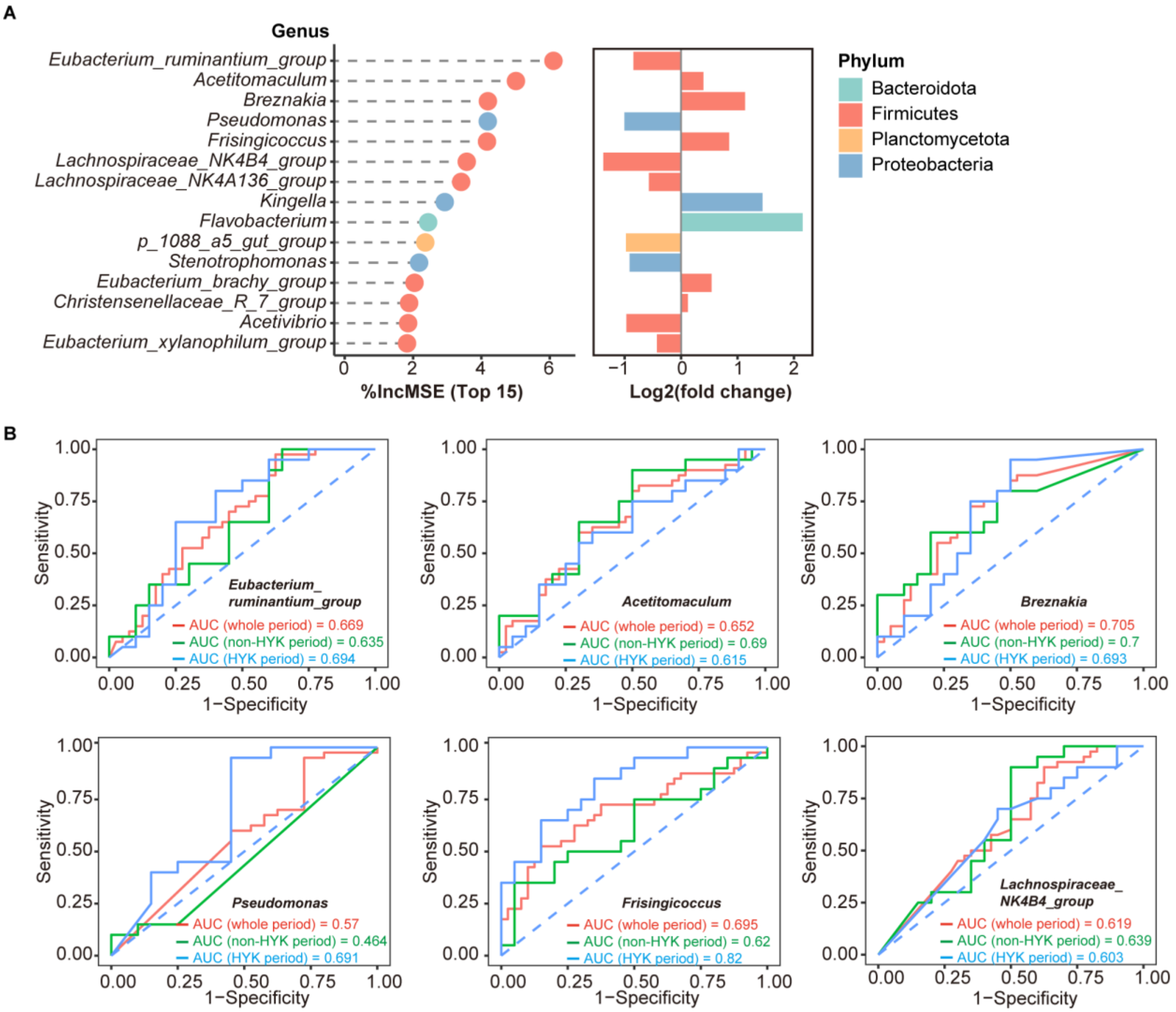

Fig S4. Key genera in development of hyperketonemia. **A** Lollipop plot shows the top15 genera ranked by mean decrease accuracy (%IncMSE) in random forest analysis. Bar plot indicates the differential abundance of genus taxa between the healthy and hyperketonemic cows during transition period. When the $\log_2$ (fold change) value is above 0, it indicates higher the abundance of that genus in the hyperketonemic cows. Conversely, the abundance of genus taxa is higher in the healthy cows if the $\log_2$(fold change) value less is than 0. **B** Receiver operating characteristic curves (ROC) were generated for important microbes (Top 6) based on random forest analysis.

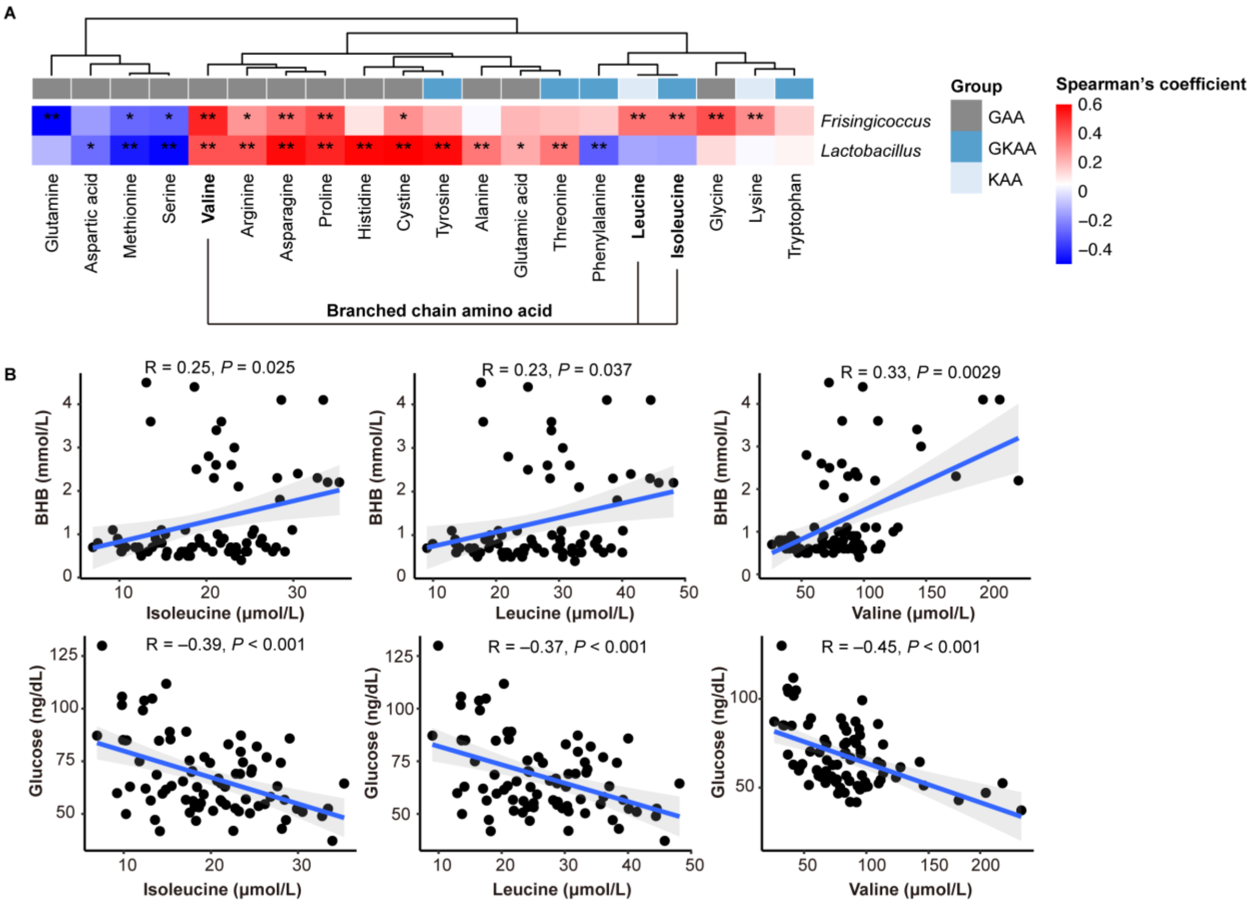

Fig S5. Associations among individual amino acid, gut microbes and serum makers. **A** The heatmap displays the correlations between individual amino acids and key genera based on Spearman's correlation analysis. $^{**}P <$ 0.01 or 0.01 < $^{*}P$ <0.05 indicates a strong correlation between two variables. **B** Branched-chain amino acids (BCAAs) participates in the ketogenesis and glycogenesis pathways. The associations among levels of BCAAs, BHB and glucose were analyzed using Spearman's rank correlation analysis.

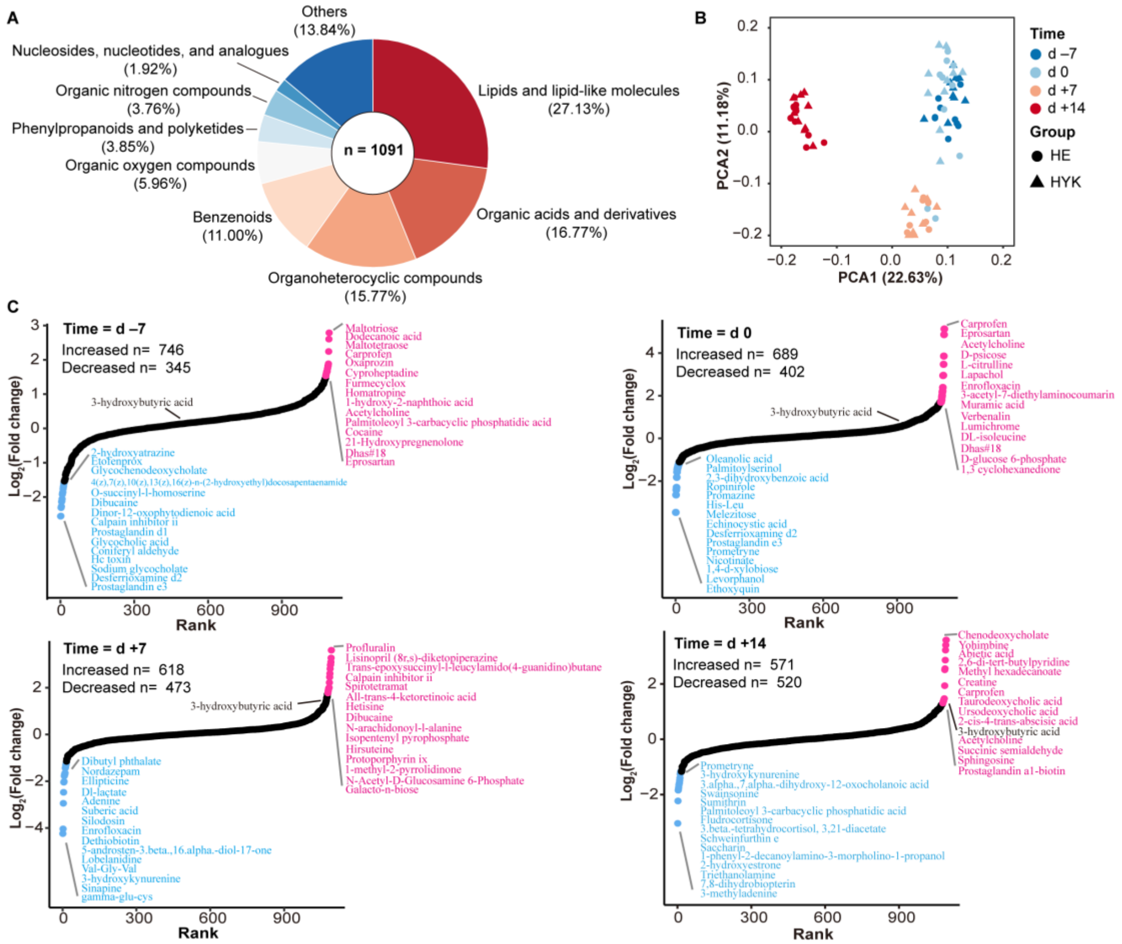

Fig S6. Fecal metabolome of dairy cows at each time point. **A** Pie chart shows the percentage distribution of metabolites by superclass. **B** Dynamic changes of fecal metabolome profiles between the healthy (HE) and hyperketonemic (HYK) groups were investigated based on principal component analysis. **C** Scatter plots indicate the result of fold change analysis for fecal metabolites between the two groups at each time point. When the $\log_2$ (fold change) value is above 0, it indicates a higher metabolites level in the HYK cows compared to the HE cows. When the $\log_2$ (fold change) value is below 0, it indicates a higher metabolites level in the HE cows compared to the HYK cows.
